# Mortality and morbidity assessment attributed to short- and long-term exposure to fine particles in ambient air of Agadir city, Morocco: The AirQ model approach

**DOI:** 10.5620/eaht.2023009

**Published:** 2023-05-12

**Authors:** Youssef Bouchriti, Amal Korrida, Mohamed Ait Haddou, Abderrahmane Achbani, Hasnaa Sine, Jamila Rida, Hayat Sine, Rachid Amiha, Belkacem Kabbachi

**Affiliations:** 1 Laboratory of Geosciences, Environment and Geomatics, Faculty of Sciences, Ibn Zohr University, Agadir, Morocco; 2 High Institute of Nursing Professions and Health Techniques of Agadir, Agadir, Morocco; 3 High Institute of Nursing Professions and Health Techniques of Agadir, Health Sciences and Environment Laboratory, Health Sciences, Epidemiology and Human Pathologies Research Team (ER-2SEPH), Agadir, Morocco; 4 Research Laboratory of Innovation in Health Sciences (LARISS), Faculty of Medicine and Pharmacy, Ibn Zohr University, Agadir, Morocco; 5 Laboratory of Cell Biology and Molecular Genetics, Faculty of Sciences, Ibn Zohr University, Agadir, Morocco; 6 High Institute of Nursing Professions and Health Techniques, Marrakech, Morocco; 7 Health Sciences Research Laboratory, Faculty of Medicine and Pharmacy, Ibn Zohr University, Agadir, Morocco; 8 Clinical Epidemiology and Medico-Surgical Sciences, Faculty of Medicine and Pharmacy, Mohammed V University, Rabat, Morocco

**Keywords:** AirQ model, Health impact, COPD, LC, ALRI, PM_2.5_

## Abstract

It is well established that respiratory mortality and morbidity are associated with high concentrations of fine particles such as PM_2.5_. The aim of this study was to evaluate the long- and short-term impacts of PM_2.5_ on the population of Agadir, Morocco, using AirQ 2.1.1 software. The mean PM_2.5_ values were obtained from data collected at three sites. Baseline incidence data were obtained from the literature, and relative risk (RR) values were referenced from the World Health Organization. This study quantified long-term total mortality (LT-TM), lung cancer mortality (LT-LC), morbidity from acute lower respiratory tract infections (LT-ALRI), and morbidity from chronic obstructive pulmonary disease (LT-COPD), as well as short-term total mortality (ST-TM). The attributable proportions (AP) of LT-TM and LT-LC were estimated to 14.19% and 18.42%, respectively. Their excess deaths were estimated to 279 and 11 persons, respectively, and their RRs to 1.16 (95% CI: 1.10-1.22) and 1.23 (95% CI: 1.12-1.37), respectively. Furthermore, the AP of LT-ALRI and LT-COPD were estimated to 14.36% and 15.68%, respectively, their excess deaths to 33 and 4, and their RRs to 1.17 (95% CI: 1.11-1.31) and 1.19 (95% CI: 1.00-1.02), respectively. In comparison, the AP of ST-TM was estimated to 1.27%, with a 25-person excess death rate. This study was conducted to inform decision-making and to promote local policies on ambient air quality.

## Introduction

The adverse effects of air pollution on health are well established and documented. Currently, about 7 million premature deaths per year are attributable to air pollution [1], and only one in ten people lives in communities that meet World Health Organization air quality guidelines (WHO AQG). Fine particulate matters are among frequently studied air pollutants that constitute major risk factors for comorbidities and have wide range of identified adverse health effects. They are defined as particles less than 2.5 µ m in aerodynamic diameter (PM_2.5_) [2], and could result from various anthropogenic or natural activities [3].

To date, strong evidence shows causal associations between exposure to PM_2.5_ air pollution and all-cause mortality, as well as mortality and morbidity from acute lower respiratory tract infections (ALRI), chronic obstructive pulmonary disease (COPD), ischemic heart disease (IHD), lung cancer (LC), and stroke [4-6]. This causality is determined in the long term for all-cause, cardiovascular and respiratory mortality [7,8]. Assuming a linear association, Chen and Hoek (2020) reported a relative risk estimate of 1.08 (95% CI: 1.06-1.09) per 10 µ g/m³ of PM_2.5_ for nonaccidental mortality [9]. Both authors evoked evidence of a supra-linear relationship implying a greater increase in risk at lower levels of exposure. Long-term effects investigated by cohort studies, revealed also associations between PM_2.5_ exposure and total, cardiovascular, and lung cancer mortality [10-13]. Sensitive groups, including the elderly, children, and patients with respiratory and cardiovascular diseases exposed to even low concentrations of air pollutants, are likely to be affected [14-17].

The finest particles are more dangerous to human health than the coarsest ones [18]. Particulate matter, primarily from combustion, is deposited throughout the respiratory tract, particularly in the pulmonary alveoli. These particles include a variety of immunogenic substances linked to asthma exacerbation symptom [19]. Particles mostly from combustion, are deposited throughout the respiratory tract in the pulmonary alveoli, and include a variety of immunogenic substances related to asthma symptoms exacerbations [20]. In addition, exposure to particles may increase the risk of neurogenerative and neurodevelopmental disorders [21, 22].

Ambient PM_2.5_ concentrations vary to a great extent between and within countries, and more than 90% of the world's population in 2019 lived in areas where concentrations exceeded the 2005 WHO air quality guideline (WHO AQG) of 10 µ g/m^3^ [23]. Likewise, low- and middle-income countries are expected to account for 91% of PM_2.5_-related mortality in outdoor and indoor air, with a per capita rate three times higher than the one of high-income countries [24]. Furthermore, in their 2017 study on premature death from changes in air pollution attributable to climate change, Silva et al. (2017) reported that PM_2.5_ would cause 55,600 (-34,300 to 164,000) deaths in 2030 and 215,000 (-76,100 to 595,000) in 2100 (countering by 16% the global decrease in PM_2.5_-related mortality). If some major pollutants, such as PM_2.5_ and NO_2_, persist at present levels, air pollution is expected to cause around 2.5 million cases of noncommunicable diseases also by 2035 [25].

In 2021, the PM_2.5_ annual WHO AQG level has been lowered from 10 µ g/m^3^ to 5 µ g/m^3^. The 24-hour WHO AQG level for PM_2.5_ changed from 25 µ g/m^3^ to 15 µ g/m^3^.

Based on the WHO AQG, Morocco's air quality is moderately affected. In 2014, the economic cost of air pollution in Morocco represented 1.05% of the country's GDP [26]. Moreover, Moroccan cities suffer from high particle air pollution as a result of vehicle traffic and industry, as well as considerable terrigenous inputs related to the aridity of the climate and proximity to the desert. However, because monitoring networks are still in their early stages, data on these cities is limited. Cities in the South are now confronted with pollution from industrial sources as well as urban traffic of polluting vehicles as a result of late industrialization. According to the most recent WHO statistics from 2016, the yearly average concentration of PM_2.5_ in Morocco is 28.38 µ g/m^3^, ranging from 23.6 to 35.6 µ g/m^3^ [27].

So far, only five studies in Morocco have been conducted to quantify the health impact of particulate matter using, in addition to PM_10_, SO_2_, NOx, and O_3_ as exposure markers [28-32].

Despite the adverse health effects associated with ambient exposure to PM_2.5_, limited information is published on the spatial and temporal variation of PM_2.5_ concentrations in Morocco, and their health effects in terms of mortality and morbidity, particularly in a metropolis like Agadir City. The objective of this study was thus, to quantify the long-term mortality and short-term morbidities associated to ambient PM_2.5_ exposure in this area.

## Materials and Methods

### Study Area

**Figure 1.**
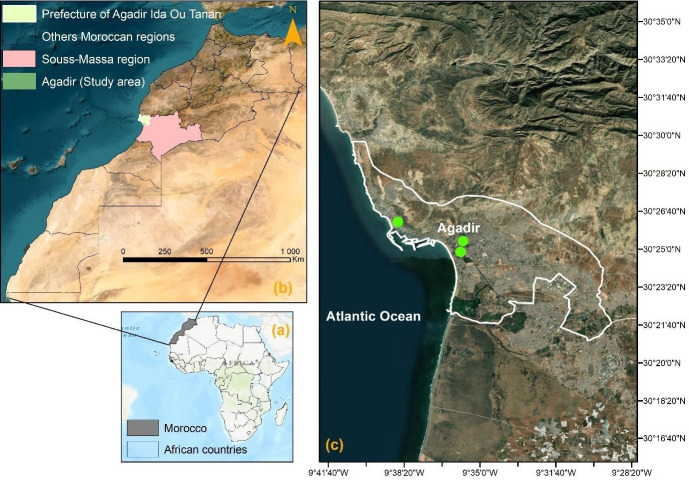
Location map of the study area: (a) Morocco in Africa; (b) Study area in Agadir Ida Ou Tanan Prefecture; (c) Study area; Locations of the mobile air pollution measuring station in 2016 (green circles)

Agadir city is located in southwest Morocco on the Atlantic coast. Its latitudinal and longitudinal coordinates, as well as altitude are respectively, 30°25′12′′ north, 9°35′53′′ west, and 31 meters above the sea level (Figure 1). The city is also the administrative capital of the Souss-Massa Region. According to the General Census of Population and Habitat in 2014 (GCPH 2014), Agadir city had a population of 421,844 persons and constitutes Morocco's first tourist destination and the country's first fishing harbor. Nevertheless, there is currently little evidence on the effects of air pollution on Agadir population. In 2020, the public transport system will have 43 lines and a fleet of 206 vehicles. The system connects the whole of the small communities, while peri-urban lines connect the agglomeration to localities up to 50 kilometers away. The annual rainfall is 250 mm. Between November and March, there are just a few days of rain. There are over 340 days of sunlight every year, however mist and morning dew are typical in the summer. The year-round trade wind front has a considerable impact on temperatures, which fluctuate little between winter and summer. In January, average temperatures vary from 14 to 16°C, while in July, they range from 19 to 22°C. However, upwellings of Saharan air occur on occasion, causing the temperature to climb beyond 40 °C for a few days. Agriculture, along with tourism, are the primary economic activities.

### Air pollution data monitoring and exposure assessment

The average PM_2.5_ concentrations in 2016 were considered to be as indicators of short- and long-term exposures among Agadir inhabitants. These data were collected from the 2016 monthly report of provided by the Regional Committee for Monitoring and Surveillance of Air Quality in Souss-Massa Region. The automated PM_10_ sensor measures the amounts of PM suspended in the ambient air instantly. The measurement basic is beta ray absorption, and the PM collecting device is made up of filtering membranes 47 mm in diameter, which can be used for mass measurement control using the gravimetric technique and/or chemical tests.

In the Souss-Massa region, there is only one mobile monitoring unit for air pollutant concentrations. This station has been set for 10 months for the first location (April to November 2016) and 2 months for the second location (May and June 2016) (green circles in Figure 1).

### Population and Health Data

The long-term health impact of PM_2.5_ is assessed considering all-cause death in adults aged 30 and older, as well as LC mortality in adults aged 25 and older. Yearly statistics obtained from the Souss-Massa Regional Hospital Center (SMRHC) on chronic obstructive pulmonary disease (COPD) cases in 2016 were used. Data on acute lower respiratory tract infections (ALRI) in children under the age of five were obtained from examinations of preschool and primary school students, during Ministry of Health campaigns conducted against transmissible ophthalmia in 2015 Health Report. Due to a lack of local data on the incidence of LC, standardized incidence of LC in 2004 was used as a reference from Casablanca Regional Cancer Registry. Finally, to compute baseline cumulative incidences of all-cause mortality and morbidity caused by ALRI and COPD, yearly statistics data from 2015 Health Report and SMRHC were used.

### Statistical analysis using AirQ+ software

To assess the burden of disease resulting from air pollution and its effects, AirQ+ software developed by the WHO Regional Office for Europe was implemented [33]. AirQ+ includes methods for assessing the short- and long-term impacts of exposure to ambient air pollution. The main methods make use of scientific data from epidemiological cohort studies that indicate associations between long-term average concentration levels in polluted air and mortality risks in exposed populations.

AirQ+ estimations are based on the application of concentration-response functions, which relate exposure levels to a pollutant with a number of health events across the study area and period. These functions represent the relative health risk associated with a certain change in exposure level. They are developed from epidemiological studies and can be used when the causal nature of the association can be accepted.

The estimated proportion of attributable cases, estimated number of attributable cases, estimated number of attributable cases per 100,000 persons at risk, attributable proportion of cases in each air pollutant concentration category, cumulative distribution by air pollutant concentration, and life years lost are the main results generated by AirQ+.

To quantify long-term and short-term effects, the following information was provided: (1) annual average air quality data for long-term exposure effects, (2) population at-risk data, such as the total number of adults 30 years of age, (3) health data, such as baseline rates of health outcomes in the Greater Agadir population, (4) a threshold value to be considered (10 µ g/m^3^ as recommended by WHO), and (5) relative risk values (RRs).

For AirQ+, the relative risk due to air pollution is usually modeled with a log-linear function [34], this RR is calculated as RR = expβ×(X-X0), where X denotes the pollutant concentration (µ g/m^3^), and X0 denotes the threshold or counterfactual value, e.g: the background concentration or lowest possible value (µ g/m^3^). In this study x0 = 10 µ g/m^3^ for PM_2.5_ (annual mean not to be exceeded according to WHO AQG 2005). In the log-linear model, β indicates the change in RR for a one-unit change in concentration x.

The attributable proportion (AP) is the attributed fraction of the health effect caused by exposure in a given population over a given time interval, and it can be calculated as AP = 1 - 1/RR(c), where RR(c) is the relative risk for the health effect in the exposure category (c).

The number of cases per unit population (BE) can be calculated using BE = B × AP and a chosen baseline incidence (B) of the extreme health effect in the population. The number of attributable cases (AC) for a population of size N can be calculated as NE = N × AP [35].

AirQ+ incorporates default values for RRs, or concentration-response functions, for many health outcomes, as well as national conversion factors between PM_2.5_ and PM_10_ and solid fuel consumption data. Only deaths reported by the SMRHC that fulfilled the requirements in the AirQ+ software were considered.

## Results and Discussion

During the study period, the annual average PM_10_ was 57.17 µ g/m^3^, and its concentration was 2.86 times higher than the 2005 WHO AQG (20 µ g/m^3^). The average PM_2.5_ concentration was obtained by converting the PM_10_ concentration according to a factor of 0.62 established by WHO in 2016. The average value of PM_2.5_ concentration after conversion was then estimated to 35.45 µ g/m^3^, and was 3.55 times higher than the 2005 WHO AQG (10 µ g/m^3^), as well as the national average concentration assessed by the WHO to 13.55 µ g/m^3^ (11.07-16.7 µ g/m^3^) [36]. The available data on annual mean PM_10_ concentrations are from 2016. The update of the WHO AQG occurred in 2021; therefore, the WHO AQG of 2005 was adopted.

The relative humidity percentage in Agadir in 2016 varied from 34.75 to 65.88%, with an average of 52.5%. The yearly averages for temperature and wind speed were 21.6 °C and 2.34 m/s, respectively.

Table 1 shows that in 2016 intra-hospital mortality was estimated to 1966 deaths. During the same year, 366 cases of ALRI in children under 5 years of age and 28 cases of COPD in adults 30 years of age were reported. The annual incidence per 100,000 persons of Total mortality, LC, ALRI, and COPD for 2016 was 968.48, 613.91, and 11.63, respectively.

### Long-term PM_2.5_ ambient air pollution and adult mortality

Table 2 shows the long-term health effects caused by PM_2.5_ (total mortality, LC mortality, ALRI morbidity, and COPD morbidity) and the short-term health effects of PM_2.5_ (total mortality). AirQ+'s default RR for all-cause mortality is estimated to 1.062 (95% CI: 1.040-1.083). According to the results in Table 2, for an annual average PM_2.5_ concentration of 35.45 µg/m^3^, 279 deaths caused by long-term exposure to PM_2.5_ would be theoretically preventable out of the 1,966 deaths in all age groups in 2016. If the PM_2.5_ concentration did not exceed 10 µg/m^3^, the AP was 14.19%. The measured RR was estimated to be 1.16 (95% CI: 1.10-1.22). In this case, the estimated RR indicates that for an annual PM_2.5_ concentration that exceeds 10 µg/m^3^, the risk of death for 30-year-olds is 1.16 higher than for the other age groups.

Recent studies have found that PM has the biggest harmful impact on human health. Although various pollutants have been associated to health effects, PM_2.5_ has been given the most attention [2].

PM_2.5_ is recognized as one of the most important environmental health risk factors due to the possibility of particle penetration (deeper into the lung) [18].

Time series studies have revealed that pollution exposure increases risk. It investigates the daily association between air pollution and emergent health impacts such as cardiovascular and respiratory disease death or hospital admissions due to cardiovascular and respiratory disease. In this research, the RR value for the increase in overall mortality induced by PM_2.5_ was 0.16% for every 10 µg/m^3^ increment in pollutant concentration.

Given the rise in RR, it is reasonable to assume that PM_2.5_ had the greatest impact on human health by increasing its concentration. This conclusion was also confirmed in other countries exposed to different degrees and levels of pollution, such as China, the USA, and India [37-39]. In addition, recent studies conducted showed that several deaths have been attributed to long-term PM_2.5_ exposure [40-43].

### Long-term PM_2.5_ ambient air pollution - integrated exposure-response functions (IER)

The IER function integrates RR information from studies on ambient air pollution, solid home cooking fuel, passive smoking, and active smoking [44]. These functions are used to estimate the disease burden associated with longterm exposure to PM_2.5_ in ambient air. AirQ+ is used to calculate the effects of PM_2.5_ on the basis of WHO air quality guidelines. Models such as log-linear or linear-log can be employed. Current IER functions have only been developed for specific adult mortality causes: IHD, stroke, COPD, and LC. Furthermore, IER functions for ALRI in 5-year-old children are estimated.

AirQ+ can also be used to assess changes in impact. Indeed, the IERs have been modified over the years to produce estimates of the Global Burden of Disease (GBD) [45]. In this regard, the counterfactual concentration range of 2.4 to 5.9µg/m^3^ was chosen to produce the 2015 GBD [6]. The values set by the 2005 WHO AQG were used to explore the benefits of reducing air pollution. Each exposure-response function refers to a specific age group.

#### Long-term ambient PM_2.5_ air pollution and adult LC mortality

The main question to be addressed here is: what are the expected health benefits (in terms of reduced LC cases) if the current level of air pollution measured by PM_2.5_ data, is reduced to the interim targets?

GBD 2015/2016 (integrated function 2016 with WHO interim target 1) was adopted as the calculation method with 2.4 µg/m^3^ as the threshold value for this model. The standardized incidence per 100,000 people is estimated for all regions in Morocco at 25.53. The population at risk is adults aged 25 years and older estimated at 240,825 according to GCPH 2014 [46].

The impact of PM_2.5_ exposure is assessed for the 2016 annual average of 35.45 µg/m^3^. Table 2 shows that lung cancer mortality is modestly decreased due to the expected low incidence, resulting in an estimated 11 avoided deaths in 2016. It's indeed important to note that the AP estimate for the current year is 18.42%.

Pope et al. (2011) calculated the adjusted associated risk of LC mortality for 10 µg/m^3^ of ambient PM_2.5_ to 1.14 (95% CI: 1.04-1.23) [47]. In a similar study, the associated risk of LC mortality from PM_2.5_ exposure was calculated to 1.14 (95% CI: 1.04, 1.23) [41]. Another study found that a 10 µg/m^3^ rise in PM_2.5_ was related with an increased incidence of LC (RR = 1.34; 95% CI: 1.10-1.65) [48]. Faridi et al (2018) found that deaths attributed to LC were 83 cases in 2015 [42].

According to Yarahmadi et al. (2018), the average annual number of LC deaths related to long-term PM_2.5_ exposure in Tehran was 142 cases [41]. Differences in baseline mortality could explain the differences in LC death values of this study compared to those above.

#### Long-term PM_2.5_ ambient air pollution and ALRI morbidity in adults

The goal is to estimate the expected health benefits in terms of reduced ALRI cases in children under the age of five if current levels of air pollution, as measured by PM_2.5_ data, are lowered to the interim targets. The analysis method used is based on the GBD 2015-2016 function (2016 integrated function with WHO 3 interim target), with 15 µg/m^3^ as the model's threshold value.

In 2015, the cumulative incidence per 1000 persons was estimated to be 613.91. In Table 2, the AP due to PM_2.5_ exposure over the model target value of 15 µg/m^3^ was estimated to 14.36% in 2016, with 33 cases of ALRI in children under the age of five potentially avoidable.

These findings are consistent with those of Sonego et al. (2015), who found that air pollution was significantly associated with an increased risk of death from ALRI [49], and Darrow et al. (2014), who reported that PM_2.5_ was associated with emergency department visits for pneumonia and upper respiratory tract infection in children under the age of five [50].

#### Long-term ambient PM_2.5_ air pollution and mortality in adults with COPD

The objective was to estimate the benefits of lowering the number of COPD cases in children and people aged 25 and older if present levels of air pollution, as assessed by PM_2.5_ data, are lowered to interim targets.

The GBD 2015-2016 function (a 2016 integrated function with the WHO 3 interim target) is used as the calculation method, considering 15 µg/m^3^ as the model's threshold value. In 2016, the cumulative incidence per 100,000 persons is estimated to 11.63.

The results of Table 2 indicate that in 2016, the AP due to exposure to PM_2.5_ above the target value taken by the model of 15 µg/m^3^ is estimated at 15.68%, while 4 of the 29 COPD cases reported by the pulmonary service of the SMRHC for adults aged 25 and over are potentially avoidable.

### Short-term PM_2.5_ ambient air pollution and adult mortality

The WHO does not propose that short-term exposure to PM_2.5_ be quantified as a substitute to long-term exposure, although it is done for guidance. We are interested in quantifying the number of deaths from non-accidental causes that are attributed to short-term daily exposure to air pollution that exceeds the 2005 WHO AQG for PM_2.5_ (25 µg/m^3^).

AirQ+'s default RR for all-cause mortality was estimated to be 1.0123 (95% CI: 1.0045-1.0201). Calculations for mortality in adults aged 30 and older were conducted, using annual PM_2.5_ exposure averages of 35.45 µg/m^3^ in 2016. As predicted, short-term impacts are minor in comparison to long-term ones.

In fact, the results indicate that excess deaths would be potentially avoidable if the WHO recommended values for PM_2.5_ were met. It should be noted that the effects of short-term exposure to PM_2.5_ are largely included in the estimates of the effects of long-term exposure.

Table 2 shows that in 2016, with an annual average PM_2.5_ concentration of 35.45 µg/m^3^, 25 out of the 1,966 deaths across all age groups might have been avoided if the PM_2.5_ concentration did not exceed 25 µg/m^3^.

The AirQ software model has some limitations. For instance, it does not consider health impacts induced by exposure to combinations of so many pollutants, their toxicities or their interactions, instead of focusing on the effect of a single pollutant.

Also, the threshold values used to calculate RRs, are relative to studies conducted mainly in Europe and/or the Unites States. Finally, estimations generated by AirQ+ software are subject to uncertainty due to concentration-response functions based on restricted assumptions and levels of evidence.

## Conclusions

To our knowledge, no research has been carried out to investigate the disease burdens associated with PM_2.5_ exposure in Agadir City, Morocco. Furthermore, in a recent systematic review on air quality in Morocco and its health impact on the Moroccan population conducted by Bouchriti et al. (2023), 1230 records were identified, of which 31 were eligible, all of which showed an exceedance of annual concentrations of air pollutants in relation to WHO guidelines [51]. Due to the reduced data from only two monitoring stations, the results of this study suggest that they should be interpreted with caution. Hence, the implementation of the AirQ+ software developed by the WHO, allowed the assessment and quantification of health effects of air pollution in this city for the first time. Based on the findings of the present study, and to help reduce air pollution, and consequently improve air quality, the following might be recommended. First, supporting research on the effects and consequences of air pollution on human health through the use of specific mobile sensors rather than the expensive fixed stations. Second, digitalizing health data in hospitals and primary health care centers to improve the information system and outcomes. Third, encouraging carpooling and developing a sustainable urban public transport system that minimizes greenhouse gas emissions. Finally, bringing national air quality standards up to date with the most recent WHO air quality recommendations.

Despite AirQ+ software limitations, the model provides a helpful and appropriate tool for leaders and health groups to boost collective awareness towards community environmental management and monitoring.

## Figures and Tables

**Table 1.** Population characteristics.

Health endpoint	Age (years)	At-risk population	Hospital cases or deaths	Annual incidence per 100,000 persons
Total mortality	≥ 30	202,999	1,966 deaths	968.48
LC	≥ 25	240,825	-	25.53 ^1^
ALRI	< 5	54,731	336 cases	613.91 ^2^
COPD	≥ 25	240,825	28 cases ^3^	11.63

1 Standardized incidence for all Morocco regions (Casablanca region cancer registry results in 2004).

2 Annual cumulative incidences calculated among students examined in preschool and elementary school as part of the Communicable Ophthalmia Campaign in 2015.

3 The SMRHC's pneumology service reported cases in 2016.

LC: Lung cancer, ALRI: Acute lower respiratory tract infections, COPD: Chronic obstructive pulmonary disease.

**Table 2.** PM_2.5_ health impact assessment results in AirQ+ in 2016.

Exposure	Outcome	Health endpoint	Age (years)	RR (CI 95%) ^1^	AP (%) ^2^	NAP ^3^
Long-term	Mortality	Total mortality ^4^	≥ 30	1.16 (1.10-1.22)	14.19 (9.5-18.37)	279 (187-361)
		LC ^5^	≥ 25	1.23 (1.12-1.37)	18.42 (11.02-26.26)	11 (7-16)
	Morbidity	ALRI ^6^	< 5	1.17 (1.10-1.24)	14.36 (8.78-19.33)	33 (20-44)
		COPD ^7^	≥ 25	1.19 (1.11-1.31)	15.68 (9.66-23.38)	4 (3-7)
Short-term	Mortality	Total mortality	≥ 30	1.01 (1.00-1.02)	1.27 (0.47-2.06)	25 (9-40)

1 Relative risk with its 95% confidence interval for a threshold value of 10 μg/m^3^.

2 Estimated attributable proportion (%).

3 Estimated number of attributable cases.

4 RR estimation by the log-linear model using formula: RR (x) = e^β×(x-x_0_)^, β = 0,006015392281974714 (0,006015392281974714 – 0,007973496801885352).

5 RR estimation method: GBD 2015/2016 (integrated function 2016 with WHO interim target 1). Threshold value: 2.4μg/m^3^.

6 RR estimation method: GBD 2015/2016 (integrated function 2016 vs WHO AGQ value). Threshold value: 10 μg/m^3^

7 RR estimation method: GBD 2015/2016 (integrated function 2016 with WHO 3 interim target). Threshold value: 15 μg/m^3^.

8 RR estimation method: GBD 2015/2016 (integrated function 2016). Threshold value: 2.4 μg/m^3^ [6].

LC: Lung cancer, ALRI: Acute lower respiratory tract infections, COPD: Chronic obstructive pulmonary disease, GBD: Global Burden of Disease.
